# Regulation of the Germinal Center Response

**DOI:** 10.3389/fimmu.2018.02469

**Published:** 2018-10-25

**Authors:** Marisa Stebegg, Saumya D. Kumar, Alyssa Silva-Cayetano, Valter R. Fonseca, Michelle A. Linterman, Luis Graca

**Affiliations:** ^1^Babraham Institute, Cambridge, United Kingdom; ^2^Instituto de Medicina Molecular, Faculdade de Medicina, Universidade de Lisboa, Lisbon, Portugal; ^3^Instituto Gulbenkian de Ciência, Oeiras, Portugal; ^4^Centro Hospitalar Lisboa Norte–Hospital de Santa Maria, Lisbon, Portugal

**Keywords:** germinal center (GC), Tfr cell, Tfh cell, immuneregulation, humoral responses

## Abstract

The germinal center (GC) is a specialized microstructure that forms in secondary lymphoid tissues, producing long-lived antibody secreting plasma cells and memory B cells, which can provide protection against reinfection. Within the GC, B cells undergo somatic mutation of the genes encoding their B cell receptors which, following successful selection, can lead to the emergence of B cell clones that bind antigen with high affinity. However, this mutation process can also be dangerous, as it can create autoreactive clones that can cause autoimmunity. Because of this, regulation of GC reactions is critical to ensure high affinity antibody production and to enforce self-tolerance by avoiding emergence of autoreactive B cell clones. A productive GC response requires the collaboration of multiple cell types. The stromal cell network orchestrates GC cell dynamics by controlling antigen delivery and cell trafficking. T follicular helper (Tfh) cells provide specialized help to GC B cells through cognate T-B cell interactions while Foxp3^+^ T follicular regulatory (Tfr) cells are key mediators of GC regulation. However, regulation of GC responses is not a simple outcome of Tfh/Tfr balance, but also involves the contribution of other cell types to modulate the GC microenvironment and to avoid autoimmunity. Thus, the regulation of the GC is complex, and occurs at multiple levels. In this review we outline recent developments in the biology of cell subsets involved in the regulation of GC reactions, in both secondary lymphoid tissues, and Peyer's patches (PPs). We discuss the mechanisms which enable the generation of potent protective humoral immunity whilst GC-derived autoimmunity is avoided.

Interactions between T and B cells are critical for the development of most humoral immune responses; these can be protective in response to vaccination or infection, or deleterious, when driving autoimmunity, allergy, or transplant rejection. Long-lived T-dependent humoral immunity is derived from specialized microanatomical structures known as germinal centers (GCs), that form in secondary lymphoid organs, such as the spleen and lymph nodes, upon infection, or immunization with a T-cell dependent antigen (Figure 1) (1). Ectopic GCs can also appear in non-lymphoid tissue in multiple inflammatory states including autoimmune disease, cancer, and during infection (2). Within GCs, B cells undergo somatic hypermutation (SHM) of the genes encoding their B cell receptor (BCR). Because this mutational process is random, mutated B cells require selection to ensure that only B cells bearing a BCR with an improved affinity for antigen differentiate into long-lived antibody secreting plasma cells and memory B cells (3). Therefore, tight regulation of GCs is critical to ensure that a potent immune response against foreign antigen can occur without cross reactivity against self-antigens.

**Figure 1:**
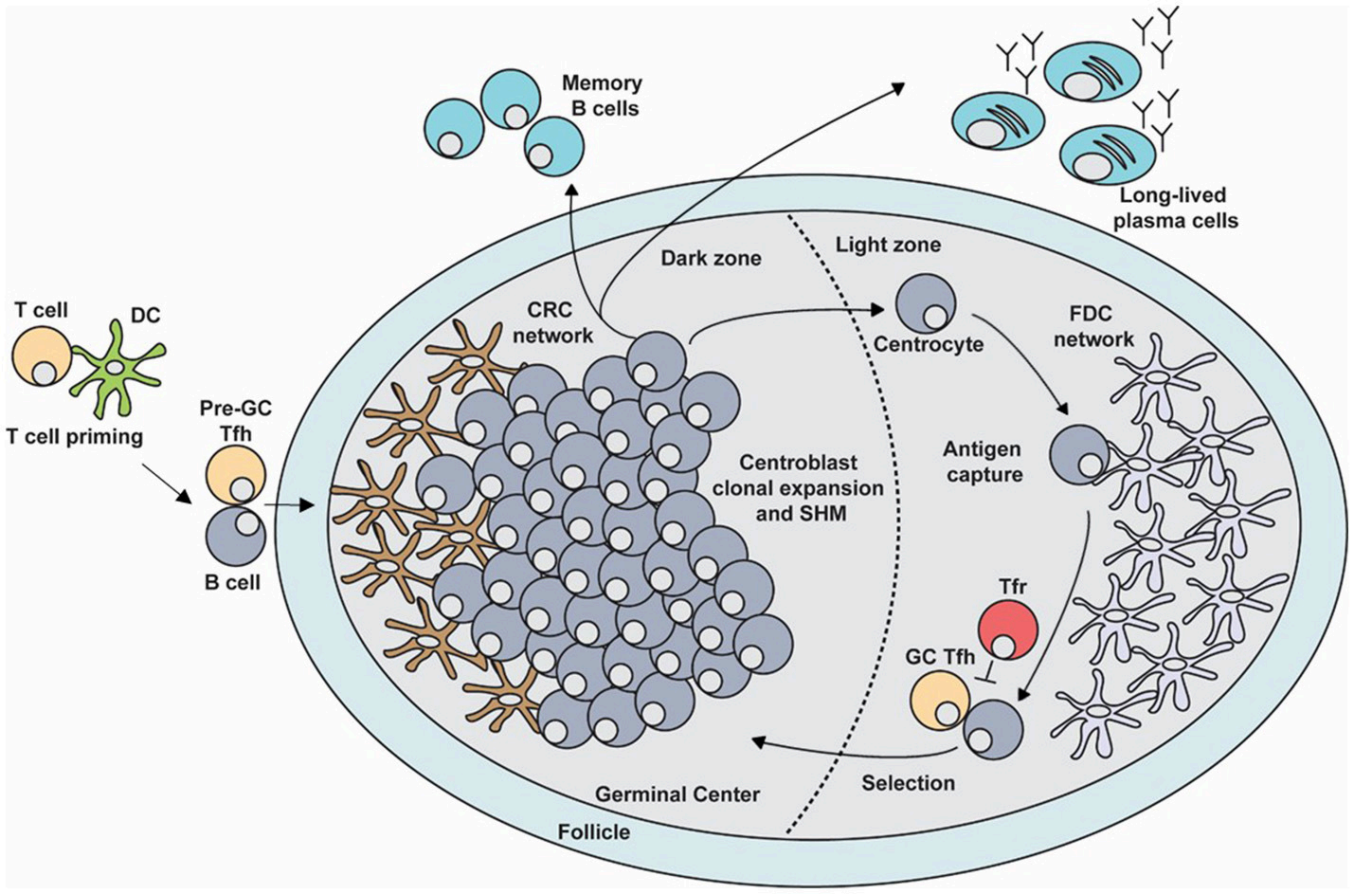
The germinal center (GC) response. The GC is a specialized microenvironment formed within the B cell follicles of secondary lymphoid tissues upon infection or immunization. The GC is divided into two distinct compartments. The dark zone (DZ) that contains a network of CXCL12-producing reticular cells (CRCs) and is the site of GC B cell proliferation and somatic hypermutation (SHM). Centroblasts then follow a CXCL13 gradient to enter the light zone (LZ) as centrocytes through their expression of CXCR5. In the LZ, centrocytes capture antigen presented on follicular dendritic cells (FDCs) which they internalize, process and subsequently present to T follicular helper (Tfh) cells in order to undergo selection. This process is regulated by T follicular regulatory (Tfr) cells which are also present in the LZ. Upon receiving survival signals from Tfh cells, centrocytes re-enter the DZ for further rounds of proliferation and SHM after which they exit the GC as memory B cells or high-affinity antibody-secreting plasma cells.

For B cells to participate in the GC response, they first need to recognize their cognate antigen via their BCR. B cells are able to directly bind soluble antigen or bind antigen presented on the surface of follicular dendritic cells (FDCs), macrophages or dendritic cells (4–7). Once activated by antigen encounter, B cells upregulate the chemokine receptor CCR7, which facilitates the migration of B cells via a chemokine gradient toward the CCR7 ligands CCL19 and CCL21 expressed in the T cell zone (8). At the interface between the B cell follicle and the T cell zone (T:B border), B cells present fragments of peptide antigen on major histocompatibility complex (MHC)-Class II to CD4^+^ helper T cells that provide them with survival and co-stimulatory signals (8, 9). B cells will then divide at the perimeter of the follicle and will either initiate the GC response or differentiate into short-lived extrafollicular plasma cells or memory B cells (10–12). Extrafollicular plasma cells produce the first wave of antibodies before undergoing apoptosis within a few days, providing an initial burst of antibodies that are essential for early control of infection while the GC response is established (13).

After cognate interactions with CD4^+^ T cells, activated B cells will migrate to the center of the follicle to seed the GC response (14). These GC B cell precursors begin to rapidly divide and undergo clonal expansion during which the GC is divided into two distinct compartments known as the dark zone (DZ) and the light zone (LZ; Figure 1) (15). The DZ contains the rapidly diving B cells known as centroblasts, which undergo SHM (16–18). Centroblasts express the chemokine receptor CXCR4 whose ligand, CXCL12, is produced by stromal cells in the DZ (CXCL12-expressing reticular cells, CRCs). This chemokine localizes the centroblasts within the DZ, thereby generating GC polarity (16, 19). Once GC B cells have undergone SHM in the DZ, they downregulate CXCR4 and migrate to the LZ, to receive positive selection signals. The LZ is rich in FDCs that produce CXCL13, which attracts GC B cells that exit the DZ as centrocytes, through their expression of CXCR5 (15, 17, 18). The LZ also contains Tfh and Tfr cells that are important for the successful and regulated continuation of the GC response (3). FDCs and Tfh cells are critical for the positive selection of centrocytes, while Tfr cells are thought to regulate the output of the GC response (3). Together, these processes culminate in the emergence of long-lived antibody secreting plasma cells and memory B cells whose BCRs bind antigen with high affinity. These effector cells are able to provide protection against subsequent infection, in some cases providing life-long immunity against particular pathogens.

## DC-initiated Tfh cell development is essential for the GC response

Tfh cells are unique in their ability to support GC reactions. Tfh differentiation is a multistage process (20). First, naïve CD4^+^ T cells are primed by dendritic cells (DCs). During these interactions, T cells require two signals to be activated: first binding of the T cell receptor (TCR) to peptide:MHC and secondly, a co-stimulatory signal through ligation of the receptor CD28 by its ligands CD80/86 which are expressed on the surface of DCs. During this T:DC interaction, T cells also integrate signals from multiple cytokines that skew their differentiation toward a Tfh cell fate. Here, Tfh cell precursors (pre-Tfh cells) upregulate Bcl6 and CXCR5, and downregulate CCR7, leading to migration of activated T cells toward the T:B border. Here, SAP-dependent interactions with activated B cells enable full Tfh cell differentiation.

It is now clear that specific subsets of DCs can support the initial steps of Tfh differentiation (21). Although it appears that this is not a “one DC fits all responses” rule as different types of immune stimuli trigger different DC populations to support Tfh cell differentiation. Adjuvants that trigger Toll-like receptor (TLR)-9 enable monocyte derived DCs to induce Tfh differentiation (22). In Th2 skewed responses, CD8a^−^ conventional dendritic cells (cDCs) are capable of inducing Tfh cell differentiation through higher expression of ICOSL and OX40L co-stimulatory signals compared to CD8a^+^ DCs in both mice and humans (23, 24). Similarly, CD11b^+^ cDC (cDC2, which are CD8a^−^) cells are both necessary and sufficient for Tfh induction following intranasal immunization (25). These cDC2 have a phenotype consistent with location at the T:B border. In contrast, CD301b^+^ DCs are thought to limit effective Tfh differentiation and antibody responses following immunization with type 2 adjuvants (26), through expression of the inhibitory costimulatory ligand PD-L1 (26). Taken together, priming of naïve CD4^+^ T cells by DCs is essential for the first step in Tfh cell differentiation, but multiple DC types are capable of doing the job.

## Regulation of the GC response by chemokines and the stromal cell network

### Chemokines and immune cell migration

**Figure 2:**
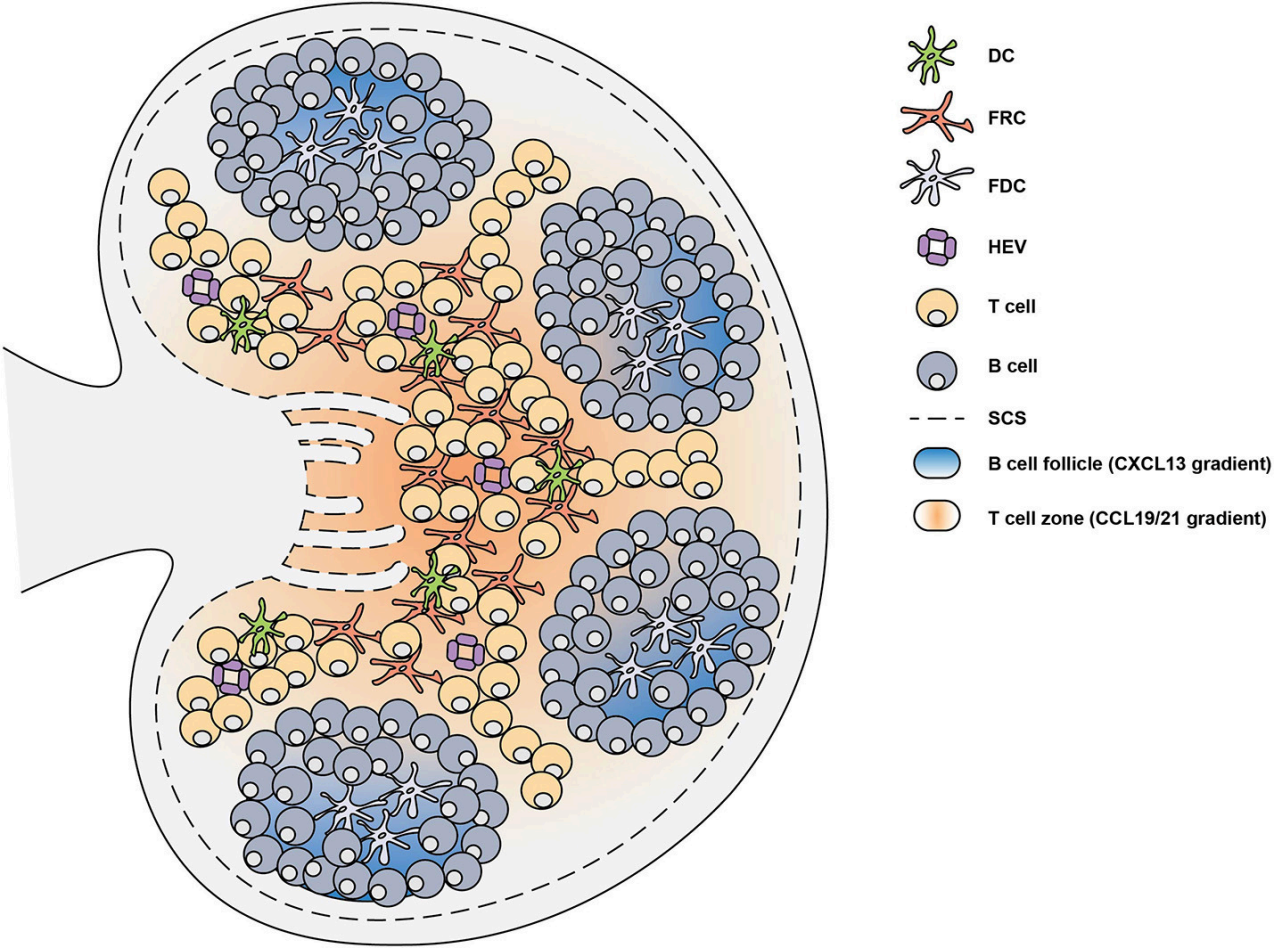
Lymph node structure is supported by stromal cells. Secondary lymphoid organs are divided into distinct regions through the generation of chemokine gradients by stromal cells. In the lymph node (LN), these chemokine gradients allow the circulation of lymphocytes which enter through high endothelial venules (HEV). In the T cell zone, fibroblastic reticular cells (FRCs) generate a CCL19 and CCL21 gradient which facilitates the migration of T cells and dendritic cells (DCs). The B cell follicles contain follicular dendritic cells (FDCs) which generate a CXCL13 gradient that promote the localization of B cells. Upon infection or immunization, antigen can enter the LN through the subcapsular sinus (SCS) or can be brought by DCs to trigger the activation of lymphocytes.

The chemokine system coordinates the migration and positioning of immune cells within secondary lymphoid organs (27). Chemokines are typically secreted chemotactic cytokines that constitute a family of more than 40 small proteins with a molecular weight of 7–12 kDa (28). Chemokines are able to mediate the migration and positioning of immune cells by engaging G protein-coupled receptors (GPCRs), expressed on the surface of all immune cells, with high affinity (29). Various lymphoid and non-lymphoid cells are able to express chemokines (29), however the expression of chemokines by mesenchymal stromal cells is critical for guiding lymphocytes and dendritic cells (DCs) to secondary lymphoid organs (SLOs) during the initiation of the immune response (27). Within the SLOs, different types of stromal cells play specific roles to facilitate the localization of hematopoietic cells (Figure 2). In the T cell zone, fibroblastic reticular cells (FRCs) orchestrate the migration, localization and survival of DCs, T cells and B cells by producing CCL19, CCL21, and CXCL12 (30–32). The CCL19/21 produced by FRCs enables localization of both CD4+ T cells and DCs to the T cell zone, via CCR7-mediated migration, bringing these rare cells together to facilitate T cell priming and activation (33–35). In the B cell follicles there are two types of stromal cells: follicular dendritic cells (FDCs) that produce CXCL13 and CXCL12-producing reticular cells (CRCs) which promote the localization of B cells during the germinal center response (19, 36, 37). FRCs at the boundary of the T cell zone and follicle produce B cell-activating factor (BAFF) to maintain the primary follicle structure (30). Antigen encounter by naïve B cells is facilitated by CXCR5-mediated migration toward the CXCL13-rich follicles (38–40). This localizes them close to the subcapsular sinus (SCS) where small soluble antigens are drained and can directly trigger B cell activation (38–40). Alternatively, antigens drained through the SCS can be captured by follicular FDCs which typically recognize antigen bound by antibody and/or complement, known as immune complexes (ICs) (41). FDCs are able to retain antigen on their surface for prolonged periods of time, allowing B cells to scan the follicular FDC network for cognate antigen to trigger activation (42). In addition, the SCS facilitates the movement of lymph fluid and is lined by marginal reticular cells (MRCs), which are FDC precursors and provide structural support (43). The distribution of these stromal cells in specific areas of the SLOs facilitates the continuous circulation and subsequent activation of lymphocytes that enter the LNs and Peyer's patches (PPs) through high endothelial venules (HEV) (44). Together, the stromal cell network provides the structural and chemotactic support required for GC initiation and maintenance.

### Stromal cells and chemokine gradients regulate GC initiation

The initiation of the GC requires both CD4^+^ T cells and B cells to be activated by cognate antigen. The initial encounter and activation of lymphocytes by antigen is facilitated by stromal cell networks, as described above. Once both CD4^+^ T cells and B cells are activated by antigen, they must migrate toward the T:B interface to undergo cognate interactions that ultimately lead to GC formation (27). Activated CD4^+^ T cells begin to downregulate CCR7 and upregulate CXCR5 which allows them to move away from the CCL19/21-rich T cell zone and toward the interfollicular region (45–47). Simultaneously, activated B cells upregulate CCR7 while maintaining CXCR5 expression which allows them to move toward the edge of the follicle at the T:B interface (48). Additionally, both cell types upregulate Epstein-Barr virus-induced G protein coupled receptor 2 (EBI2) which facilitates their localization at the T:B border (49–51). Stromal cells at the inner- and outer-follicle regions regulate the oxysterol ligands for EBI2 which facilitates the co-localization of EBI2^+^ T and B cells enabling cognate T:B interactions (8, 51–54).

The final step for GC formation requires activated CD4^+^ T cells and B cells to migrate to the follicle from the T:B border as GC B cells and fully differentiated Tfh cells. Both CD4^+^ T cells and B cells downregulate CCR7 and EBI2 whilst stably expressing CXCR5. This enables them to escape the chemotactic pull of the T cell zone and outer follicle in order to move into the center of the follicle (14, 51, 55, 56). Both CD4^+^ T and B cells upregulate S1P receptor 2 (S1PR2) that supports their localization to the follicle center by binding S1P, which is present in low concentrations within the follicle, and reduces their responsiveness to other chemoattractants (57, 58). Loss of S1PR2 results in B cells losing their ability to accurately localize at the center of the follicle and the combined loss of S1PR2 and CXCR5 abrogates T cell localization to the GC (57, 58). At the center of the follicle, signals exchanged between T and B cells provide the final cues for Tfh cell differentiation and promote B cell proliferation to seed the GC (59). Once the GC response is initiated, both the FDC and CRC networks expand and divide the GC into the two distinct light and dark zones. Both the CRC and FDC networks are essential in maintaining the function and structure of the GC while orchestrating the interactions between different immune cells of the GC.

### The role of follicular stromal cells in the regulation of the GC response

The network of CRCs was recently discovered due to their high expression of CXCL12 in the DZ and they were found to have low network density as well as a net-like morphology (19, 37). These cells are distinct from FDCs and FRCs as they do not express the typical FDC/FRC markers which include CD35, ERTR7, FDC-M1/M2, FcγRII, and VCAM1 (37). Due to the lack of antigen-capture mediators such as CD35 and FcγRII on the surface of CRCs, it is likely that CRCs do not function as antigen presenting cells but meet another specialized requirement in the DZ niche. Thus, CXCL12 production is believed to be one of the essential functions of CRCs in the GC DZ (27). Moreover, two-photon laser-scanning microscopy revealed that GC B cells are able to crawl in and around CRC networks, which depend on CXCR4 signaling for their distribution (37). Therefore, CRCs likely provide support for GC B cells through structural maintenance of the DZ in addition to generating a CXCL12 gradient within the GC. However, the precise role of CRCs in the GC remains to be fully elucidated.

In contrast to the CRCs, FDCs were discovered in the 1960s and are better characterized. During GC formation, the expansion of FDCs is mainly thought to be driven by proliferation of MRCs and their subsequent differentiation into FDCs (60). Throughout this process the FDCs also become activated through TLR4 (61, 62) and B-cell derived lymphotoxin (LT) α1β2 signaling (63), though the precise mechanisms remain unidentified. Once activated, FDCs begin to increase their expression of CXCL13 and BAFF, which support GC development and maintenance of the LZ (27). Studies in mice have shown that ablation of FDCs results in GC termination due to reduced survival and localization of GC B cells; therefore FDCs are absolutely necessary for the GC response (64). The activation of FDCs also triggers an increase in their expression of antigen-capture molecules such as CD35, CD21, and FcγRII (27). These molecules are critical for the long-term retention and display of antigen on the surface of the FDC network. This allows antigen-specific B cells to test their Ig receptors by capturing antigen from FDCs to subsequently internalize, process and present the antigen peptides to Tfh cells in order to receive critical survival signals (17, 42). FDCs can also produce cytokines such as IL-6 (65, 66) and IL-15 (67, 68) that promote SHM and IgG production and support B cell proliferation, respectively. Additionally, FDC production of FDC-M1 aids in the clearance of apoptotic GC B cells as FDC-M1 coats B cells, marking them for clearance by tangible-body macrophages (69).

### Stromal cells regulate Tfh and GC B cell interactions within the GC

The CRC and FDC networks form distinct niches critical for the structural support and maintenance of the GC. These two stromal cell subsets are also crucial for the localization of GC B cells within the GC and form a spatially segregated stage where T and B cells can undergo crucial interactions to promote the generation of high-affinity antibody-secreting plasma cells and memory B cells. During the GC response, GC B cells shuttle between the DZ and LZ using a timed program (19). GC B cells can localize in the DZ through their expression of CXCR4 in response to CXCL12 (16). However as they proliferate they downregulate CXCR4 and upregulate CXCR5, which together with the FDC-mediated CXCL13 gradient, enables them to move toward the LZ (16, 19). Migration to the LZ is necessary for centrocytes to acquire antigen and present it to Tfh cells in order for high-affinity B cell clones to survive (3). Through signals received in the LZ, a subset of centrocytes is then able to re-express CXCR4 and migrate back to the DZ via CXCL12-mediated migration where they can undergo further rounds of proliferation and SHM (19). This results in bidirectional B cell trafficking between the two zones that allows for multiple rounds of proliferation and selection to further refine the affinity of responding GC B cells (17, 19).

Tfh cells are conventionally thought to localize to the LZ through their high-expression of CXCR5. For Tfh cells to be retained in the GC they must express not only S1PR2, but also SLAM-associated protein (SAP), which promotes antigen-specific T-B adhesion (70, 71). SAP-deficient T cells are able to localize to the follicle through their expression of CXCR5, but once in the follicle they exhibit severe defects in GC recruitment and retention (71). Additionally, GC retention of Tfh cells can be mediated by expression of a class B Ephrin, EFNB1, which negatively controls Tfh cell retention and also promotes interleukin (IL-21) production (72). While these studies have investigated mechanisms by which Tfh cells are retained within the GC, the functional importance of their localization within the GC compartments remains largely unexplored. GC Tfh cells are able to co-express both CXCR4 and CXCR5 (73). The expression of CXCR4 by Tfh cells has been shown to determine their localization between the LZ and DZ (73). Moreover, *in vitro* studies with human immune cells isolated from tonsils have shown FDCs may play a role in modulating CXCR4 expression on T cells (74). Another study also showed that Tfh cells which express IL-21 have high expression of CXCR4 and are able to localize closer to the DZ (75). However, the functional significance of differential CXCR4 expression of Tfh cells and their localization within the GC remains unknown largely due to the importance of CXCR4 in thymic maturation of T cells (76). Thus, GC stromal cells also play a role in directing the localization of Tfh cells.

Chemokine secretion by the stromal cell networks of SLOs is essential for the regulation of various aspects of the immune system, ranging from the homeostatic migration of lymphocytes to the initiation and maintenance of the GC response. Within the GC reaction, stromal cells provide chemokine cues that promote B cell trafficking between the different GC compartments as well as supplying antigen crucial for affinity maturation. However, whether the different stromal cell subsets of the GC can regulate the function of Tfh cells remains to be explored. Further study into the mechanisms by which stromal cells can regulate the GC will lead to a better understanding of the events required for optimal GC responses against infection and vaccination.

## Regulation of GC responses by T follicular regulatory cells

While the specialized formation of the GC and T—B cell crosstalk are critical to provide protection against a broad range of invading pathogens, the stochastic nature of SHM makes the generation of cross-/self-reactive B cell clones a by-product of GC responses to foreign antigens (77). This can lead to the development of autoimmune disease. The importance of Treg cells for the control of both autoimmune and antibody responses has been long known (78–81). Mice and humans with loss-of-function mutations in the Foxp3 gene do not form Treg cells and suffer from a fatal early-onset T cell-dependent, lymphoproliferative disorder manifested by autoantibody-mediated autoimmunity (diabetes, thyroiditis, haemolytic anemia) and increased levels of circulating antibodies (82–86). The link between antibody production and Treg cells lead researchers to identify a subset of Treg cells that gain access to the B cell follicle and participate in the regulation of the GC response (87–89). These T follicular regulatory (Tfr) cells simultaneously express markers of Treg and Tfh cells and have suppressive function (87–91). Since their discovery, Tfr cells have been regarded as putative key GC regulators that fine tune the response.

### Tfr cell differentiation

Tfr cells are derived from Foxp3^+^ precursors; the majority come from thymic Treg cells, but they can also arise from naïve T cells when immunization conditions favor induced Treg development (92, 93). The differentiation of Tfr cells is not characterized as well as the differentiation of Tfh cells, but it appears that they also undergo a multistep Bcl-6-dependent differentiation process like Tfh cells. Like other naïve CD4^+^ T cells, antigen presentation by DCs is required for Tfr cell differentiation (88, 92, 94, 95), along with positive co-stimulatory signals through CD28 and ICOS (59, 96–101). However, the DC subsets directly responsible for stimulating Tfr cell differentiation remain unclear. The differentiation into GC Tfr cells is also dependent on B-cell interactions (88, 94). However, B cells appear to be required only for final stages of Tfr cell differentiation, as putative Tfr cells were found in the blood of μMT mice following immunization and B-cell deficiency patients (BTK deficiency) (94, 102).

Despite some similarities, there are also differences in the differentiation requirements of Tfr and Tfh cells. The negative co-stimulatory molecules PD-1 and CTLA-4 impact Tfr cell generation. PD-1 signaling selectively inhibits thymic Treg cell differentiation into Tfr cells, prior to B-cell interactions in a PD-L1-dependent manner (100), while blockade of PD-L1 signals in the periphery inhibits the generation of induced Tfr cells (92). Deletion of CTLA-4 leads to an increase in the frequency and absolute numbers of Tfr cells (103, 104). However, it is still unknown whether CTLA-4 impairs Tfr cell differentiation or maintenance, or whether the increased Tfr cell numbers are simply due to an increased GC response overall. IL-21 is a key helper cytokine produced by Tfh cells, which has a negative impact on Tfr cell numbers (105, 106), suggesting that Tfh cells evoke a feedback mechanism to control Tfr cell numbers via this cytokine (107, 108). Mechanistically, Jandl and colleagues propose that IL-21 induces Bcl-6 expression which in turn limits CD25, and the reduction of CD25 expression then leads to lower responsiveness to IL-2, consequently restraining Tfr cell expansion (105). However, because it has been shown that Tfr cells do not express CD25, the high affinity IL-2 receptor(109–111), it is more likely that IL-21 would limit Tfr cell precursors, rather than fully differentiated Tfr cells. CD25 expression limits Tfr differentiation through induction of Blimp-1 (109), a transcription factor known to repress Tfr differentiation (88). Although, IL-2 seems to inhibit Tfr cell differentiation, the absence of IL-2/STAT5 signaling may lead to Foxp3 downregulation (112). Therefore, the maintenance of Foxp3-expressing Treg cells in the absence of IL-2/CD25 (IL-2Rα) must be accomplished by other homeostatic mechanisms, such as high amounts of intermediate-affinity IL-2 receptor (CD122/IL-2Rβ), which may be sufficient to prevent Foxp3 downregulation (109). Whether CD25^+^ and CD25^−^ Tfr cells represent two stages of differentiation or two functionally and biologically distinct cell subsets is still unknown.

Tfr cell differentiation culminates in the expression of Bcl-6, the master transcriptional regulator for Tfr cell differentiation (87–89). However, it is not the only transcription factor that contributes to the Tfr cell fate. Expression of transcription factor nuclear factor of activated T cells 2 (NFAT2, also known as NFATc1) is required for CXCR5 upregulation on Treg cells through binding to the *Cxcr5* promoter (113). Tfr cell differentiation also requires an intricate network of many other molecules, such as stromal interaction molecule 1 (STIM1) and STIM2 (114, 115), tumor necrosis factor receptor (TNFR)-associated factor 3 (TRAF3) (116), signal transducer and activator 3 (STAT3) (117), p85α-osteopontin, and members of the helix-loop-helix family (E and Id proteins). More recently, mTORC1 signaling was also shown to induce *de novo* Tfr cell differentiation from thymic-derived Treg cells (118).

### Identity and specificity of Tfr cells

The first studies describing Tfr cells reported that these cells are derived from thymic Treg cells (87–89). Recent evidence further supports the preferential origin of Tfr cells from thymic Foxp3^+^ Treg cells (93), but Tfr cells can also be derived from induced Treg cells, that arise from the induction of Foxp3 expression in naïve CD4^+^ T cells (92). Tfr cell recruitment into the GC and their suppressive capacity occurs in response to immunization, but Tfr cells do not need to be specific for the immunizing antigen (93, 94). However, a proportion of Tfr cells can also be specific for the immunizing antigen (92), suggesting that Tfr cells can arise though a number of pathways. TCR repertoire analysis at the population level showed that Tfr cells are an oligoclonal population and have a TCR repertoire that is more similar to the repertoire of Treg cells than Tfh cells (93, 119). How Tfr cells are recruited to the GC upon immunization, with TCR specificities that are irrelevant to the immunizing antigen, is a significant unknown in the biology of these cells.

One remaining question regarding the regulation of humoral immunity is the overall contribution of extrafollicular responses to the generation of B cell autoreactivity and its regulation. It is not clear to which extent extrafollicular sites contribute to the emergence of autoreactivity in many diseases, and it remains unknown whether specialized regulatory mechanisms are in place at those locations.

### Mechanisms of Tfr cell function

**Figure 3:**
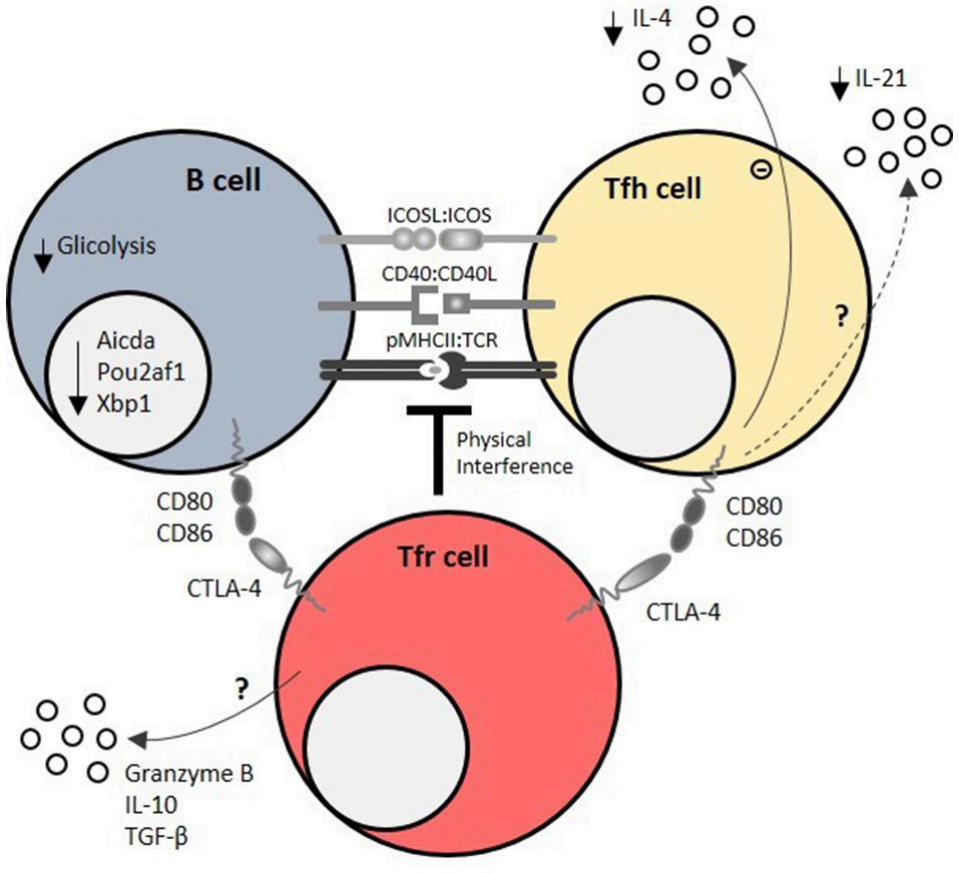
Mechanisms of Tfr cell-mediated regulation of humoral responses. Tfr cells regulate T – B interactions within the germinal center (GC) by physical interference at the immunological synapse, which is required for the survival feedback loop between GC B cells and T follicular helper (Tfh) cells. CTLA-4 is a key molecule of T follicular regulatory (Tfr) cell function at immunological synapse, as it directly blocks CD80/CD86 co-stimulatory signals. Using these mechanisms, Tfr cells impair GC B cell metabolism (mainly by decreasing glucose uptake and usage) and induce the downregulation of GC B cell effector molecules, such as Pou2af1 (required for GC B cell formation), Xbp1 (required for antibody secretion), and Aicda (required for class switch recombination). On the other side of the immunological synapse, Tfr cells limit IL-21, and IL-4 secretion by Tfh cells. Granzyme B, IL-10 and TGF-β secretion by Tfr cells may also account for their regulatory capacity.

Tfr cells specialize in the regulation of the GC response by directly modulating Tfh cell proliferation, B cell metabolism and cytokines secreted by Tfh cells in secondary lymphoid organs. Thus, Tfr cells modify GC outcomes at several levels: (a) control of GC size; (b) selection of antigen specific Tfh and B cell clones; and (c) modulation of class switch and affinity maturation of antibodies (Figure 3).

The precise molecules that underpin such effects are largely unknown, but undoubtedly encompass CTLA-4-mediated suppression (103, 104). CTLA-4 has a widely known function in maintaining immune homeostasis and mediating Treg cell function (81, 120–122). Mice that lack CTLA-4 on Treg cells have spontaneous GC responses (104), however in these experiments the caveat is that CTLA4 is lost on all Treg cells, not only Tfr cells. Tamoxifen-induced CTLA-4 depletion on Treg cells at the time of immune challenge led to an expansion of GC B, Tfr, and Tfh cells due to defective Treg cell function (103). Whether CTLA-4 mediated CD80/CD86 transendocytosis plays a role in Tfr cell function within GCs is still controversial (103, 104). Despite the described role of CTLA-4 in mediating Tfr cell function, it is expected that these cells employ multiple and complementary regulatory mechanisms such as TGF-β, IL-10 (88, 94) and granzyme B secretion (88, 123) (Figure 3) to perform their suppressive functions.

The suppressive functions of Tfr cells seem to act directly on the metabolic pathways of B and Tfh cells (94, 100, 103, 106). Using several *in vitro* systems, it was shown that Tfr do not affect the transcriptomic signature or activation potential of B cells or Tfh cells, however these cells lose the ability to express key effector molecules, such as Pou2af1, Xbp1 and Aicda, in the presence of Tfr cells (106). It appears that Tfr cell-imposed B cell modulation persists in the absence of Tfr cells due to epigenetic changes. However, IL-21 was able to overcome the suppressive effect of Tfr cells by both increasing B cell metabolism and inhibiting Tfr cells (106).

While initial studies ascribed the control of GC size, class-switching and antibody affinity to Tfr cells (87–89, 124), more recent studies support the concept that Tfr cells can restrain the generation of antigen-specific antibodies while favoring the emergence of high affinity antibody-secreting B cells (103, 104, 109, 124, 125). Indeed, CTLA-4 competent Tfr cells seem to impose more stringent B cell competition for Tfh cell help (103, 104). Additionally, the outcome of the GC reaction can also be correlated with age-induced alterations in Tfh and Tfr cell numbers and function (126). In aged mice, reduced titers of NP-specific antibodies following NP-OVA immunization were associated with defective Tfh cell function and higher proportions of highly suppressive Tfr cells (126).

These regulatory mechanisms have been further studied in murine models of autoimmune disease, where Tfr cells were directly implicated in ensuring tolerance to self-antigens and preventing autoimmunity (109, 113, 115, 127). Thus, the selective use of Tfr cells (or IL-2 to fine-tune Tfr cell responses) might be a novel way to therapeutically intervene in diseases where pathogenic GC reactions are the cause of underlying pathology.

Taken together, it appears that cognate interactions are required for Tfr-mediated regulation, while the specific nature of the cellular interactions are yet to be fully characterized. Results from *in vitro* experimental systems devoid of any antigen-presenting cells besides B cells suggest that B-Tfr interactions can trigger regulation that is sufficient to overcome the positive signals delivered by co-cultured Tfh cells (106).

### Division of labor between Treg and Tfr cells

Although we have a good understanding of broad Treg cell biology, it is still unclear how different Treg cell subsets integrate to underpin immune tolerance and regulation of humoral responses. It is clear that the absence of Foxp3^+^ Treg cells leads to uncontrolled and spontaneous humoral responses (128–131), however, the contribution of CXCR5^+^ Tfr cells to this overall pathology is not known. Nevertheless, humoral suppressive capacity has been assigned preferentially to Tfr cells. This concept arose from observations where conventional (non-Tfr) Treg cells lacked the capacity to suppress Tfh cell proliferation, B cell activation, and class switch recombination (94, 106). Conversely, two independent groups found a comparable decrease in Tfh cell proliferation when co-cultured with Tfr and conventional Treg cells (87, 104). Hence, while a direct comparison of Tfr and conventional Treg cells in physiological conditions is lacking, Tfr cells presumably acquire their unique humoral suppressive capacity when they co-opt the Tfh cell differentiation program. The suppression of Tfh cell proliferation is probably not unique to Tfr cells, as it might be a general Treg cell feature. However, one critical aspect that might distinguish Tfr from conventional Treg cells *in vivo* is the exceptional ability of Tfr cells to access the GC.

### Blood and human Tfr cells

GC reactions are orchestrated in secondary lymphoid organs, but in the blood of mice circulating Tfr cells-like cells have also been described (94). This adds an additional layer of complexity, as different immune compartments might evoke different Tfr cell responses (94, 100, 132–135). The population of circulating ICOS^lo^ Tfr cells were shown to behave as memory cells and have less suppressive capacity. They originate after priming by DCs, but without full commitment to the GC fate (94, 100). This suggests that Tfr cell effector activity is initiated during contact with DCs in the T cell zone, strengthened in the inter-follicular region during contact with B cells, and optimized in the GC. However, it is not clear where exactly Tfr cells modulate GC reactions, especially in humans. Recently, human Tfr cells were found to be preferentially distributed at the periphery of GCs (136). While the same has also been shown in murine models (100), it is still unknown whether human Tfr cells share all the biological features of murine Tfr cells. For instance, in human lymph nodes, Tfr cells are not PD-1^+^CD25^−^ like in mice (100, 109, 136).

Although a CD69^−^ human tonsil Treg cell subset with B cell suppressive function was discovered before the identification of Bcl-6^+^Foxp3^+^ Tfr cells in mice (137, 138), the restricted access to human secondary lymphoid tissues forced the search for putative Tfr cells in human blood. Several studies have focused on circulating CXCR5^+^Foxp3^+^ T cells to define Tfr cells in humans with different diseases (90). We recently established the biology and ontogeny of human blood CXCR5^+^Foxp3^+^ Tfr cells (102). Human blood CXCR5^+^Foxp3^+^ Tfr cells comprise Tfr cell precursors arising from secondary lymphoid tissues prior to B-cell interactions. Thus, human blood Tfr cells are predominantly CD45RO^−^ naïve cells not yet endowed with full B cell and humoral regulatory functions. Furthermore, autoimmune diseases may be associated with different types of dysregulation of the GC response. Therefore, it is likely that different alterations of Tfr frequency, distribution, and function will be found in different autoimmune diseases.

### Peyer's patches: specialized germinal centers in a unique anatomical location

GCs in Peyer's patches (PPs) are unique due to their special anatomical location and functions. They are influenced by the gut microbiota, and in return produce IgA antibodies which contribute to the control of gut microbial homeostasis. Due to their special environment and function, these GCs require specialized forms of regulation (139).

Peyer's patches are non-encapsulated lymphoid tissues associated with the small intestinal epithelium. In mice, 6–12 PPs are interspersed along the whole length of the small intestine, while the human intestine is associated with 100–200 PPs (139). PPs are continuously exposed to antigenic stimulation by the commensal microbiota. The intimate cross talk with the gut microbiota is what sets PPs apart from other lymphoid tissues. The gut microbiome is a complex mix of bacteria, fungi, viruses and protozoa, which populates the whole intestine. Constant stimulation through this microbiota drives the formation of constitutively active GCs in PPs. These GCs produce antibodies against infectious pathogens, but also generate commensal-specific IgA antibodies that promote homeostasis of the gut microbiome (140).

### PPs as places for TD IgA production

PPs are an important site for T cell dependent IgA production (139). Like other GCs, B cells within PP GCs undergo somatic hypermutation of the Ig locus, followed by selection of B cells bearing BCRs that bind antigen with high affinity. One key difference to peripheral LNs, however, is that in PPs class-switch recombination (CSR) to the IgA isotype occurs (141). IgA antibodies exist as dimers and are secreted at all mucosal surfaces. In the gut this is mediated by M cells in the sub-epithelial dome of PPs. Once in the gut, IgAs bind to a wide variety of commensal bacteria and alter the composition of the microbiome through a variety of mechanisms (140). These include blocking antigen interactions with the host, trapping antigens in the intestinal mucus or interfering with invasive properties of pathogens (140). In addition, IgA antibodies assist with the controlled intestinal uptake of bacterial antigens to boost antigen-specific gut immune response (142, 143). In AID-deficient animals that lack CSR and SHM, there is aberrant expansion of anaerobic gut commensals and extensive immune hyperplasia (144, 145). Patients with selective IgA deficiency also exhibit changes in their gut microbiome, associated with increased Th17-cell associated inflammation (146). This demonstrates the key role that switched antibody responses play in gut health.

What is not clear is whether this IgA needs to come from the GC response. Evidence suggesting this is not the case comes from studies in which mice lack either T-dependent immune responses (CD28-deficient mice and CD40-deficient mice) or Tfh cells (*Bcl6*^flox/flox^ *Cd4*^cre/+^). These animals have high IgA antibody titers, near-to-normal levels of bacterial IgA coating, and relatively normal composition of the microbiota (147–149). However, SHM of IgA antibodies mainly occurs in GCs and analysis of mice that express a variant of AID that can facilitate CSR, but not SHM, revealed that this strain exhibited aberrant expansion of commensal bacteria and increased bacterial translocation into mesenteric LNs (150). This suggests that GC responses in the PP can play an important role in the maintenance of microbial homeostasis.

### Immune regulation of GCs in PPs

Given the distinct architecture and location of PPs, their regulatory mechanisms are unique from those in lymph node GCs. Most importantly, Tfh and Tfr cells in PPs are responsive to modulation by the gut microbiota. The ensuing plasticity in T cell regulation allows PP GCs to respond adequately to intestinal infections or changes in the gut microbiota.

#### Immune regulation of PPs by Tfh cells

PPs provide a unique environment for Tfh cell differentiation, where the “rules” established for Tfh cell development are frequently broken. Exclusively in the gut, Tfh cells can derive from RORgt^+^ Th17 cells (151) and Foxp3^+^ Treg cells (152). The precise mechanism for this is unclear, but it may be driven by stimuli from the microbiota, as microbial sensing plays an important role for Tfh differentiation in the gut. As such, the Th17 cell-promoting segmented filamentous bacteria (SFB) were shown to drive the differentiation of PP Tfh cells. Further, microbial ATP controls Tfh cell differentiation in PPs via interactions with the ATP-gated ionotropic P2X7 receptor (153). The egress of these “unusual” PP Tfh cells into systemic sites can have dire consequences for health, as they were reported to exacerbate the auto-antibody responses in arthritis (154). This demonstrates the ability of intestinal Tfh cells to integrate multiple signals from the gut microbiota for their development, with implications not only for gut, but also systemic immunity. Therefore, control of Tfh cell development, and their maintained residence in the gut is critical for organismal health.

#### Immune regulation of PPs by Tfr

Similar to Tfh cells, PP Tfr cells have gut-specific features. In PP GCs there is an increased Tfh/Tfr ratio compared to peripheral GCs (155), making the PP resemble early stages of a GC. This has been proposed to enable the expansion of low affinity B cell clones early in the response (156). This is consistent with the proposal of Reboldi et al. (139) who suggest that GCs in PPs resemble the early stages of a GC in order to favor the quick generation of diverse low-affinity antibodies in response to microbial antigens. Interestingly, gene expression profiling of Tfr cells from PPs and pLNs revealed the surprising finding that PP Tfr cells express the helper cytokine IL-4, unlike LN Tfr cells (157). This could point to a different, potentially less suppressive, role of Tfr cells within PPs.

As discussed above, Tfr cells are considered to be negative regulators of the GC response, but the data about their functionality in PPs is not clear. STAT3-KO mice, which lack Tfr cells, but have PP Tfh cells, have no observable changes in PP GC size or IgA production in the gut (117). However, in an adoptive transfer model Kawamoto et al. implicated Tfr cells in the regulation of IgA-mediated control of the gut microbiome: Supplying T cell-deficient hosts with Treg cells increased IgA production and induced dramatic changes in the composition of the microbiota (125). This is consistent with the observation that depletion of Treg cells results in a drop in IgA levels (158). Together, this suggests that both Tfr functionality as well as the Tfh/Tfr ratio in PPs are adjusted to allow for optimal control of the gut microbiota, although further work is required to precisely define the role for Tfr cells in PPs.

### Regulation of PPs by the microbiota

The gut microbiota is a crucial, but often underappreciated, regulator of the GC response in the gut and the systemic immune system. Germ-free mice, which lack any form of bacterial colonization, exhibit evident deficits in the maturation of their gut associated lymphoid tissues, including PPs and mesenteric lymph nodes. Their PPs are small and produce limited amounts of IgA antibodies (159). In addition, these mice are more susceptible to enteric infections and their systemic immune response to infections is also stunted (160, 161). This demonstrates a strong dependency of the immune system on the microbiota. There is evidence that some bacteria and their products directly affect the GC response in PPs. Transfer of a diverse microbiota into wild-type mice increases GC B cell numbers as well as bacterial IgA-coating (125). Bacterial products can also directly act on immune cells in the PP. Microbial ATP controls Tfh cell differentiation (153) and short-chain fatty acids, a diverse group of bacterial metabolites, were shown to boost plasma cell differentiation and intestinal antibody production in PPs (162, 163). This demonstrates the strong impact of the microbiota on the GC response. Thus, the interplay of the immune system with the microbiota cannot be neglected when studying the regulation of intestinal GCs.

## Conclusion

The importance of the GC response for humoral immunity has been known for several decades. However, the cellular and molecular mechanisms that regulate GC function are still being elucidated. This review highlights several known mechanisms by which GCs are regulated through the collaboration of multiple cell types in both LNs and PPs. Given the participation of GCs in physiological and pathological immune responses, a better understanding of GC regulation is likely to have clinical applications. In this respect, it is fundamental to consider and further characterize the complex cellular network and interplay that ultimately control the outcome of GC responses in specific anatomic locations. Further elucidation of the mechanisms which govern GC regulation will be beneficial to improve patient stratification in immune-mediated diseases, and for the identification of novel therapeutic biomarkers.

## Author contributions

All authors listed have made a substantial, direct and intellectual contribution to the work, and approved it for publication.

### Conflict of interest statement

The authors declare that the research was conducted in the absence of any commercial or financial relationships that could be construed as a potential conflict of interest.
